# Trends in Payer Type for Emergency Department Visits in California, 2011-2019

**DOI:** 10.1001/jamanetworkopen.2023.10321

**Published:** 2023-04-27

**Authors:** Renee Y. Hsia, Madeline Feldmeier, Nandita Sarkar

**Affiliations:** 1 Department of Emergency Medicine, University of California, San Francisco; 2 Philip R. Lee Institute for Health Policy Studies, University of California, San Francisco; 3 National Bureau of Economic Research, Cambridge, Massachusetts

## Abstract

This cohort study investigates trends in the association between emergency department use and insurance coverage in California between 2011 and 2019.

## Introduction

Emergency departments (EDs) are a crucial source of health care in the US. The past decade has brought about major changes in ED use with the introduction of the Patient Protection and Affordable Care Act (ACA), which has substantially increased the number of Medicaid-insured residents and decreased the percentage of uninsured California residents.^1^ Previous studies have analyzed trends in ED visits by insurance coverage,^2^ but to our knowledge no study has reported visit rates by payer in California, the state with the largest nonelderly uninsured population before the implementation of the ACA.^3^

Our cohort study investigates trends in the association between ED use and insurance coverage in California between 2011 and 2019 to uncover how changes in insurance have shaped the ED patient landscape. We hypothesized that visits and visit rates for patients with Medicaid would increase, offset by a corresponding decline in visits for uninsured patients, given the dramatic shift in insurance coverage and limited outpatient capacity.

## Methods

This retrospective analysis of ED visits to all nonfederal hospitals across California from 2011 to 2019 used public ED visit data from the California Department of Healthcare Access and Information and population data from the State Health Access Data Assistance Center. The study followed the Strengthening the Reporting of Observational Studies in Epidemiology (STROBE) reporting guideline, and the University of California, San Francisco (UCSF) institutional review board approved the study with a waiver of informed consent because the study used deidentified data that did not constitute human participants research, in accordance with UCSF Human Research Protection Program guidelines and 45 CFR §46.

Our analysis included all ED visits, regardless of whether the visit resulted in hospital admission or discharge. Visits were grouped into 5 categories by payer: Medicare, Medicaid, private insurance, uninsured, or other. Visit rates were determined using California population data stratified by insurance coverage. The analysis was conducted using Stata statistical software version 16 (StataCorp) and R statistical software version 4.1 (R Project for Statistical Computing). We used Spearman nonparametric rank correlation test to determine any significant trends in visit rates per year by payer and the Wilson’s score method to determine 95% confidence intervals. A 2-sided *P*  < .05 was considered to be significant for this study.

## Results

The overall number of ED visits in California grew steadily from 11 960 452 visits in 2011 to 14 853 783 visits in 2019 (24.2%), outpacing population growth (37 638 369 million people in 2011 to 39 512 223 million people in 2019 [5.0%]). Visits covered by Medicaid increased by 91.0%, from 3 230 217 visits in 2011 to 6 171 273 visits in 2019, comprising 41.5% of all California ED visits in 2019. The total number of Medicaid beneficiaries grew from 7 226 719 beneficiaries in 2011 to 9 926 000 beneficiaries in 2019 (37.4%), accounting for 25.1% of California's population in 2019. Uninsured visits decreased by 48.6%, from 1 882 072 visits in 2011 to 967 420 visits in 2019. Visits covered by Medicare increased by 37.7% from 2 469 572 visits in 2011 to 3 400 426 visits in 2019, whereas the number of visits covered by private insurance was stable over this period (Table).

**Table.** Total Population and Number of ED Visits by Payer, 2011-2019

Insurance type	Year								
	2011	2012	2013	2014	2015	2016	2017	2018	2019
Population, No.									
Medicaid	7 226 719	7 364 347	7 678 033	8 894 000	10 272 000	10 576 000	10 383 000	10 324 000	9 926 000
Medicare	4 780 267	4 902 339	5 150 217	5 370 000	5 593 000	5 778 000	5 887 000	5 948 000	6 100 000
Private	21 827 704	22 657 002	22 693 668	23 665 000	24 138 000	24 428 000	24 824 000	24 897 000	24 913 000
Uninsured	7 424 817	6 787 453	6 500 179	4 767 000	3 317 000	2 844 000	2 797 000	2 826 000	3 002 000
Other	1 103 931	1 169 459	1 222 176	1 258 000	1 266 000	1 294 000	1 280 000	1 332 000	1 263 000
Total^a^	42 363 438	42 880 599	43 244 273	43 954 000	44 586 000	44 920 000	45 171 000	45 327 000	45 204 000
ED visits, No.									
Medicaid	3 230 217	3 362 590	3 628 916	4 856 934	5 856 603	6 270 720	6 308 598	6 116 456	6 171 273
Medicare	2 469 572	2 642 161	2 728 853	2 838 797	3 015 664	3 109 446	3 278 787	3 300 265	3 400 426
Private	3 682 089	3 749 350	3 635 656	3 781 370	3 792 434	3 735 885	3 831 744	3 820 034	3 841 096
Uninsured	1 882 072	1 892 657	1 910 267	1 387 490	1 094 222	992 897	958 205	943 311	967 420
Other	696 502	760 085	814 204	515 195	494 167	439 075	446 758	445 207	473 568
Total	11 960 452	12 406 843	12 717 896	13 379 786	14 253 090	14 548 023	14 824 092	14 625 273	14 853 783

Abbreviation: ED, emergency department.

^a^ The sum of the total populations for each individual payer group does not equal the total California population for the respective year due to some individuals having multiple insurance plans.

Considering both the number of ED visits and population size, overall California ED visit rates increased from 318 visits per 1000 population to 376 visits per 1000 population (18.3%; 95% CI, 16.8% to 19.8%; *P* = .001) from 2011 to 2019, with the highest rate (377 visits per 1000 population) observed in 2017. The ED visit rate for Medicaid increased from 447 visits per 1000 population to 622 visits per 1000 population (39.1%; 95% CI, 36.9% to 41.2%; *P* < .001) from 2011 to 2019, the largest increase in rate across all payer groups over this period (Figure). Uninsured visit rates increased from 253 visits per 1000 population to 322 visits per 1000 population (27.1%; 95% CI, 20.6% to 33.9%; *P* = .025) from 2011 to 2019. Medicare rates increased from 517 visits per 1000 population to 557 visits per 1000 population (7.9%; 95% CI, 5.5% to 10.3%; *P* = .008), whereas privately insured visit rates decreased from 169 visits per 1000 population to 154 visits per 1000 population (−8.6%; 95% CI, −5.9% to −11.2%; *P* = .003). In 2019, the Medicaid ED visit rate was the highest among all groups (622 per 1000 population), even higher than Medicare (557 per 1000 population). The uninsured visit rate was 322 per 1000 population, and the privately insured visit rate was the lowest at 154 per 1000 population.

**Figure.**
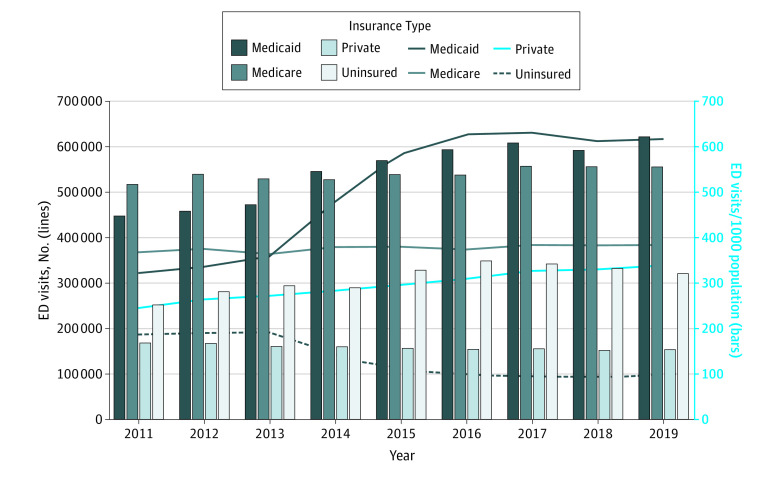
Emergency Department (ED) Visits and Visit Rates by Payer, 2011-2019 Graph shows changes in ED visits and visit rates by payer between 2011 and 2019. The lines depict changes in overall ED visits by payer; bars depict changes in ED visits per 1000 population by payer.

## Discussion

Findings from this cohort study of ED visits by payer revealed that ED visits outpaced population growth from 2011 to 2019. The number of Medicaid beneficiaries grew more than any other payer group over the study period (37.4%) and accounted for the majority of ED visits and the largest increase in visit rate. Despite comprising only 25.1% of California’s population in 2019, Medicaid visits accounted for 41.5% of all ED visits. As a result, EDs are now facing the dual impact of these changes: a larger Medicaid population and increased rates of ED use.

Although Medicaid expansion has provided increased health care coverage for many previously uninsured patients, prior literature^4,5^ suggests that health care utilization and public spending increase with government insurance coverage. Furthermore, the high number of saturated physician panels in the US continues to create barriers to accessing primary care.^6^ Our analysis is limited in that we used administrative data, which may not be generalizable outside of California. These findings may help guide policy makers and health care stakeholders when determining health care resource allocation.

## Notes

Supplement.
Data Sharing Statement
