# Supplementary material for: Trends in Payer Type for Emergency Department Visits in California, 2011-2019
Source: JAMA Netw Open. 2023 Apr 27;6(4):e2310321. doi: 10.1001/jamanetworkopen.2023.10321 (PMC10140801; doi:10.1001/jamanetworkopen.2023.10321)
Supplement: Supplement. — Data Sharing Statement [file jamanetwopen-e2310321-s001.pdf]

Data Sharing Statement

Hsia. Trends in Payer Type for Emergency Department Visits in California, 2011-2019. *JAMA Netw Open*. Published April 27, 2023. doi:10.1001/jamanetworkopen.2023.10321

Data
Data available: No
